# Effects of 5D built environment and non-built-environment factors on injury crash risk: An interpretable machine learning analysis

**DOI:** 10.1371/journal.pone.0353205

**Published:** 2026-07-07

**Authors:** Wenfang Li, Xingchen Zhang, Sen Cao

**Affiliations:** 1 School of Intelligent Transportation and Intelligent Construction Engineering, Huanghe Jiaotong University, Jiaozuo, China; 2 School of Traffic and Transportation, Beijing Jiaotong University, Beijing, China; Tongji University, CHINA

## Abstract

To identify the key determinants of traffic injury risk and clarify the relative roles of built environment factors and crash-context factors, this study develops a traffic injury risk identification framework that integrates 5D built environment variables with non-5D factors, using traffic crash data collected in Changsha, Hunan Province, China, from 2017 to 2019. After data cleaning, screening, and spatial matching, a total of 9,743 valid samples were obtained, and injury occurrence in a crash was defined as a binary dependent variable. On this basis, three feature sets were constructed, including a 5D feature set, a non-5D feature set, and a combined 5D + non-5D feature set. Random Forest (RF), eXtreme Gradient Boosting (XGBoost), and Categorical Boosting (CatBoost) models were then developed and compared, and the best-performing model was further interpreted using the Shapley Additive Explanations (SHAP) method. The results showed that the combined 5D + non-5D feature set consistently outperformed the models using either the 5D or non-5D feature set alone, indicating that traffic injury risk arises from the joint influence of the built environment and immediate crash-context conditions. Among the three models, CatBoost achieved the best performance under the combined feature set and produced the highest receiver operating characteristic-area under the curve (ROC-AUC) value, demonstrating superior overall discriminative ability. The SHAP results further revealed that, within the combined CatBoost model, 5D variables accounted for a larger share of the model-based contribution than non-5D variables. However, given the relatively small improvement in accuracy after adding 5D variables, this finding should be interpreted as evidence of complementary explanatory information rather than as a dominant source of predictive performance. Distance to the nearest metro station, lighting condition, road network density, point of interest (POI) mix, and distance to the nearest bus stop were identified as the most influential factors. Further dependence analysis showed that a greater distance to the nearest metro station generally increased traffic injury risk, whereas higher road network density and greater POI mix were generally associated with lower injury risk. From the perspective of the interaction between 5D built environment characteristics and non-5D factors, this study reveals the multidimensional pathways through which traffic injury risk is shaped. The findings also confirm the effectiveness of the CatBoost–SHAP framework for traffic injury risk identification and interpretation, and provide empirical support for urban traffic safety risk assessment, road environment optimization, and refined governance strategies.

## 1. Introduction

Road traffic injury has long been recognized as a central issue in global public health and transportation safety governance. It not only causes substantial casualties and socioeconomic losses, but also poses serious challenges to the equity, sustainability, and resilience of urban transportation systems. Compared with analyses that focus solely on crash frequency, examining whether a crash results in injury and the severity of that injury provides a more direct understanding of the actual safety consequences of transportation system operations and the underlying mechanisms of risk exposure [1]. Existing studies have shown that injury outcomes are not determined by any single factor; rather, they arise from the combined effects of vehicle operating conditions, roadway geometric characteristics, traffic control facilities, environmental context, and individual traveler attributes [2–6]. Therefore, identifying the key determinants of injury risk from the perspective of crash consequences, rather than crash counts alone, is of considerable importance for the development of more targeted road safety interventions.

With the acceleration of urbanization and the continuous restructuring of urban spatial form, the role of the built environment in traffic safety research has received increasing attention. Traditional traffic safety studies have mainly treated road safety control measures, intersection configurations, roadway alignment, and operational traffic characteristics as the primary explanatory factors. More recent research, however, suggests that built environment attributes—such as land-use intensity and mix, street network form, destination concentration, and public transport accessibility—may indirectly or directly influence traffic crashes and their injury outcomes by altering travel demand, exposure patterns, conflict opportunities, and operating speeds [7–12]. Particularly at the urban scale, traffic safety is no longer merely an issue of roadway engineering; it is also closely associated with urban spatial organization, land-use configuration, and the micro-level streetscape environment [8,9].

Against this background, the 5D built environment framework—Density, Diversity, Design, Destination accessibility, and Distance to transit—provides a well-established analytical perspective for systematically characterizing urban spatial structure. Although the 5D framework was originally developed to explain travel behavior, walking propensity, and land use–transport interactions, its application in traffic safety research has expanded in recent years. Relevant studies have shown that variables such as intersection density, street connectivity, land-use mix, the clustering of commercial and public service facilities, and proximity to transit stops may all be significantly associated with crash risk among pedestrians, cyclists, and general road users [10–17]. At the same time, studies focusing on vulnerable groups such as children and older adults have indicated that the effects of the built environment are often characterized by substantial heterogeneity. Under different age structures, activity patterns, and modes of road use, both the direction and magnitude of the effects of environmental variables on safety outcomes may vary considerably [13–16].

Despite these advances, several limitations remain in the existing literature. First, many studies on injury-related crashes have concentrated on multiclass severity classification or on specific crash types, whereas relatively limited attention has been paid to the binary outcome of whether a crash causes injury, despite its clear practical relevance for safety management [1–6]. Second, although a considerable body of evidence has accumulated regarding the relationship between the built environment and traffic safety, many studies still remain at the level of overall crash frequency and have not fully clarified the pathways through which built environment characteristics affect injury outcomes. In addition, the distinction between traffic exposure and crash risk itself is often insufficiently addressed [8,9]. Third, prior studies have tended to emphasize built environment attributes alone, while relatively few have incorporated them into a unified analytical framework together with non-built-environment contextual factors such as weather, lighting conditions, pavement surface status, road type, and traffic control measures. As a result, the formation mechanisms of traffic injury risk have not yet been comprehensively understood [2,5,6,12]. Fourth, although machine learning methods have been increasingly applied in traffic safety research and have improved predictive performance, the “black-box” nature of these models has also become more evident. Achieving both strong predictive capability and sufficient interpretability has therefore emerged as an important research challenge in this field [18–24].

It is worth noting that recent studies integrating machine learning with interpretable analytical techniques have begun to demonstrate new methodological advantages. Several studies have employed eXtreme Gradient Boosting (XGBoost), predictive analytics, and heterogeneity modeling to identify factors associated with traffic injury risk, suggesting that nonlinear relationships, variable interactions, and unobserved heterogeneity are widespread in injury-related crash research [18–24]. In built environment studies, street-view imagery, multiscale spatial features, nonlinear modeling, and spatial heterogeneity analysis have also been gradually introduced into traffic safety research, highlighting the complexity arising from the joint effects of macro-scale spatial structure and micro-scale street environments [18–21]. Overall, however, studies that focus on the binary outcome of injury occurrence, jointly examine 5D built environment factors and non-built-environment contextual factors, and further identify key influence pathways through interpretable machine learning remain relatively limited.

Accordingly, this study takes crash injury occurrence as the outcome of interest and develops an integrated variable system that includes both 5D built environment factors and non-5D contextual factors, with the aim of systematically identifying the key determinants of traffic injury risk. Methodologically, machine learning models are employed to conduct binary classification, and interpretable analytical techniques are further introduced to reveal the relative importance and directional effects of individual variables on injury risk. From the perspective of the interaction between urban spatial environment and traffic operating context, this study seeks to provide empirical support for traffic injury risk identification, refined traffic safety governance, and people-oriented roadway environment optimization.

## 2. Literature review

Research on traffic injury outcomes has traditionally focused on injury severity classification, identification of injury-related factors, and screening of high-risk scenarios. Early studies demonstrated that road traffic injury outcomes are significantly associated with roadway conditions, traffic environment, vehicle characteristics, and behavioral factors, and that the determinants of injury outcomes exhibit strong contextual dependence across regions and crash types [1]. Subsequent studies further examined traffic injury risk from the perspective of the combined effects of multiple factors, showing that variables such as vehicle type, driver distraction, intersection control type, traffic flow conditions, and collision form may all alter the likelihood of injury occurrence [2–6]. In particular, studies on pedestrian crashes have emphasized the high vulnerability of non-motorized and unprotected road users in collisions, suggesting that injury outcomes in pedestrian-related crashes are more likely to be shaped jointly by roadway environment and traffic control conditions [6].

From a methodological perspective, early studies of traffic injury risk primarily relied on Logit, Probit, Ordered Logit/Probit, and related extensions to identify statistically significant factors and interpret the direction of their effects [1–6]. With the expansion of data availability and a deeper understanding of crash mechanisms, researchers gradually recognized that injury outcomes often exhibit pronounced nonlinearities, spatiotemporal instability, and unobserved heterogeneity, which limit the ability of traditional parametric models to capture complex interactions [3–5]. For example, some studies adopted joint modeling approaches that simultaneously considered crash frequency and severity in order to improve the identification of high-risk road segments [4]. Others employed random-parameter models and bivariate ordered models to capture heterogeneous effects in angle crashes or motorcycle crashes [23,24]. These findings suggest that traffic injury risk is not a static or homogeneous outcome, but rather a probabilistic event shaped by multiple interacting conditions.

In recent years, machine learning methods have been increasingly introduced into traffic injury research. Existing studies have shown that predictive analytics and ensemble learning models offer notable advantages in injury outcome identification, particularly in handling high-dimensional variables, nonlinear relationships, and complex interactions [2,18,22]. For instance, XGBoost has been applied to the analysis of crash injury severity and has demonstrated stronger classification performance than some conventional statistical models [22]. However, while machine learning methods improve predictive accuracy, they also raise challenges related to model interpretability. Although previous studies have attempted to address this issue through sensitivity analysis, heterogeneity modeling, and related approaches [23,24], predictive accuracy alone is insufficient from the perspective of traffic safety governance. Greater attention must also be paid to identifying which factors matter most and through what pathways they influence traffic injury risk.

Beyond model performance and interpretability, it is also necessary to clarify the potential pathways through which 5D built environment factors affect injury risk. Recent studies suggest that the safety effects of compact and mixed-use urban environments are not necessarily unidirectional. Sung et al. [25], for example, evaluated the effects of 5D measures on pedestrian crashes in Seoul and found that compact and mixed-use environments may be associated with a higher risk of pedestrian crashes, while also showing a lower risk of fatal pedestrian crashes. This finding indicates that 5D factors may simultaneously increase exposure and conflict opportunities while reducing crash impact severity through lower vehicle speeds and more urbanized traffic environments. Similarly, studies on intersection safety have shown that built environment and streetscape characteristics around intersections can significantly affect traffic accident risk, highlighting the importance of conflict concentration, road design, and local spatial context in shaping crash outcomes [26].

The mechanisms linking point of interest (POI) mix, destination accessibility, and transit accessibility to injury risk can therefore be understood through several interrelated pathways. First, higher POI mix and stronger destination accessibility may attract more pedestrians, cyclists, transit users, and motor vehicles, thereby increasing activity intensity and potential conflict exposure. Evidence from studies on urban vibrancy and taxi travel demand shows that POI density, land-use mix, and transit-related built environment variables can significantly reshape the spatial distribution of urban activities and motorized travel demand [27,28]. Second, transit accessibility may influence road-user composition and modal structure. Research on metro station areas and station-level transit use has shown that 5D factors, such as population density, destination attractiveness, intersection density, and distance to intermodal connections, significantly affect transit ridership and station use [29,30]. Related studies on walking behavior, bike-sharing–metro integration, and commuting mode choice further suggest that built environment characteristics can promote walking, cycling, and transit-related trips, thereby changing the composition and interaction patterns of road users [31,32,33].

Accordingly, variables such as POI mix and transit accessibility may affect injury risk through competing mechanisms. On the one hand, they may increase crash exposure by concentrating travel activities and generating more interactions among heterogeneous road users. On the other hand, they may reduce the probability that a crash results in injury by encouraging lower-speed local travel, reducing automobile dependence, and reflecting more mature infrastructure and traffic management conditions. Therefore, the relationship between 5D built environment factors and traffic injury risk should not be interpreted simply as a direct positive or negative effect. Rather, it should be understood as the net result of multiple pathways, including exposure generation, conflict concentration, modal composition, operating speed, and infrastructure provision. This mechanism-based perspective also provides a theoretical basis for the use of interpretable machine learning methods to examine nonlinear and heterogeneous effects in injury risk analysis.

### 2.1. Progress in research on the relationship between the built environment and traffic safety

The relationship between the built environment and traffic safety has become an important and rapidly growing topic at the intersection of transport geography, urban planning, and road safety research. Stoker et al. [7] provided one of the earlier systematic reviews of pedestrian safety and the built environment, indicating that land use, roadway cross-sectional design, intersection form, pedestrian facilities, and traffic control conditions may all affect pedestrian safety. Subsequently, Saha et al. [8] proposed a conceptual framework for understanding how the built environment influences traffic safety, arguing that the built environment does not directly cause crashes but instead affects safety outcomes through intermediary mechanisms such as traffic exposure, conflict frequency, and operating speed. Merlin et al. [9] further pointed out that one key reason for inconsistent findings in the literature is the failure to distinguish between increased exposure and elevated risk per unit of exposure. In other words, although crashes may be more frequent in high-density and mixed-use areas, this does not necessarily imply that the risk per trip or per traveler is higher in those areas.

Empirical studies have examined the relationship between built environment factors and traffic safety across different spatial scales and user groups. Dai et al. [10], using network GIS analysis, found a significant spatial correspondence between built environment characteristics and pedestrian crash hotspots. Yin and Zhang [11] further incorporated D variables commonly used in walkability research and found that the number of intersections and pedestrian environment attributes exhibited spatially varying effects on pedestrian injury, indicating that the association between the built environment and pedestrian safety is not simply linear. Asadi et al. [12], in a study of urban areas in the Netherlands, showed that land-use density, functional mix, destination proximity, and road network characteristics jointly influenced crash probability, frequency, and severity. Their findings provide strong support for introducing the 5D framework into traffic safety analysis.

Studies focusing on specific population groups have further demonstrated that the safety effects of the built environment are highly heterogeneous. Amiour et al. [13], in a systematic review of children’s objective and perceived traffic safety, reported that factors such as road facilities around schools, traffic flow conditions, intersection density, and continuity of walking space affect not only children’s actual injury risk but also the subjective safety perceptions of both children and parents. Lee et al. [14] and Gálvez-Pérez et al. [15], in studies of older pedestrians in Seoul and Madrid, respectively, found that sidewalk width, signal density, and certain land-use and socioeconomic characteristics were closely associated with older pedestrian crashes, and that these mechanisms differed from those observed in the general population. Rothman et al. [16], in a multicity Canadian study, further showed that child pedestrian and cycling injuries are strongly associated with the combined effects of built environment and social environment factors, suggesting that a purely roadway-engineering perspective is insufficient to fully explain child traffic injuries.

In addition, studies focusing on specific spatial units have indicated that the effects of the built environment are also local and scale-sensitive. Okaidjah et al. [17], at the intersection level, found that the urban built environment and average income jointly influence intersection safety performance. Such findings suggest that the spatial distribution of traffic safety is shaped not only by road infrastructure, but also by the broader socio-spatial structure of cities. Therefore, examining injury crashes from the perspective of the 5D built environment is valuable not only for systematically organizing built environment variables at the theoretical level, but also for explaining differences in traffic injury risk through the logic of urban spatial organization.

### 2.2. Micro-scale streetscape, spatial heterogeneity, and explainable analysis

With the development of multisource data and computational methods, research on the built environment and traffic safety has gradually moved beyond conventional areal-unit-based statistical analyses toward more fine-grained, dynamic, and interpretable analytical approaches. On the one hand, technologies such as street-view imagery and semantic segmentation have enabled researchers to extract micro-scale streetscape characteristics at the road-segment level, including greenery visibility, building frontage, parking occupancy, and street openness, thereby allowing a more refined characterization of how street environments affect crash risk. Hu et al. [18] used street-view imagery to identify street-level built environment features and showed that fine-grained streetscape information can complement the limitations of conventional land-use and road network indicators in crash research. On the other hand, the integration of spatiotemporal hotspot analysis with geographically weighted models has enabled researchers to identify the spatial nonstationarity of built environment effects on crash trends. The coupled spatiotemporal hotspot trend analysis and geographically weighted logistic regression proposed by Qu et al. [19] demonstrated that the effects of built environment factors on crash evolution vary significantly across space.

At the same time, the adoption of nonlinear analysis and machine learning has further advanced methodological innovation in this field. Ling et al. [20] found that street network structure and land-use characteristics generally have threshold effects and nonlinear relationships with crash density, and that these relationships vary with regional population age composition and income structure. Wu et al. [21], from a multiscale perspective, showed that streetscape features, POI characteristics, and road attributes contribute differently to crash risk, and that clear interactions exist among these variables. Correspondingly, in traffic injury research, Wu et al. [22] and Delen et al. [2] demonstrated that ensemble learning and predictive analytics can improve the identification of injury outcomes, while Se et al. [23] and Fang et al. [24] showed that even within parametric modeling frameworks, temporal instability and unobserved heterogeneity remain important issues that cannot be ignored.

Overall, the existing literature provides three important insights for the present study. First, research on traffic injury risk should move beyond simple crash frequency analysis and treat crash consequences as a central explanatory focus [1–6]. Second, the effects of the built environment on traffic safety are systematic, spatially differentiated, and heterogeneous across user groups, and the 5D framework offers a useful theoretical basis for organizing related variables [7–17]. Third, the mechanisms underlying traffic injury risk are characterized by substantial nonlinearity and interaction, and therefore require machine learning and explainable analytical methods to improve both identification accuracy and practical interpretability [18–24]. Nevertheless, a review of the existing literature indicates that relatively limited attention has been paid to studies that take injury occurrence as a binary outcome, systematically integrate 5D built environment factors with non-built-environment contextual factors, and further examine their mechanisms through explainable analysis. The present study is conducted in response to this research gap and seeks to provide a more interpretable identification and analysis of traffic injury risk from the coupled perspective of urban spatial environment and traffic operating conditions.

## 3. Data and methods

### 3.1. Study area and data source

**Fig 1:**
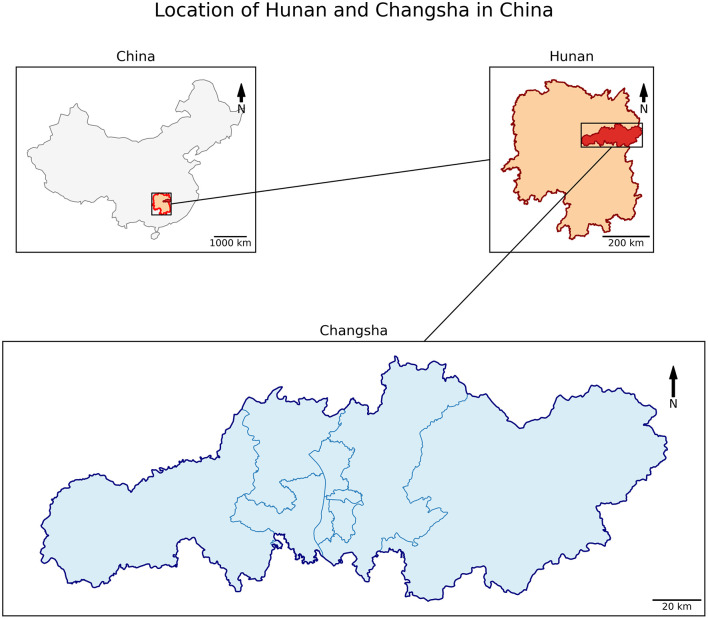
Geographic location of Changsha. Administrative boundary data were obtained from the GADM database, version 4.1 (https://gadm.org), which permits the use of its data to create maps for academic research articles, including articles published under open licenses such as CC BY. The map was created by the authors.

Changsha, Hunan Province, China, was selected as the study area (Fig 1). Changsha, located in central China, is the provincial capital of Hunan and a core city within the Changsha–Zhuzhou–Xiangtan urban agglomeration. As an important regional transportation hub, the city has a complex road network and intensive traffic activity. Marked differences are observed between the central urban area and peripheral zones in land development intensity, functional mix, public transport accessibility, and roadway operating conditions. Such spatial and transportation heterogeneity provides an appropriate setting for investigating the relationships among built environment characteristics, roadway contextual factors, and traffic injury risk.

The data used in this study were obtained from road traffic crash records in Changsha from 2017 to 2019. The original database contained information on crash time and location, road conditions, traffic control type, environmental conditions, and crash outcomes. In accordance with the objectives of this study, the raw crash records were processed through variable screening, duplicate removal, missing-value treatment, and spatial variable matching. After data cleaning and integration, a total of 9,743 complete and valid samples were retained for subsequent modeling analysis.

**Fig 2:**
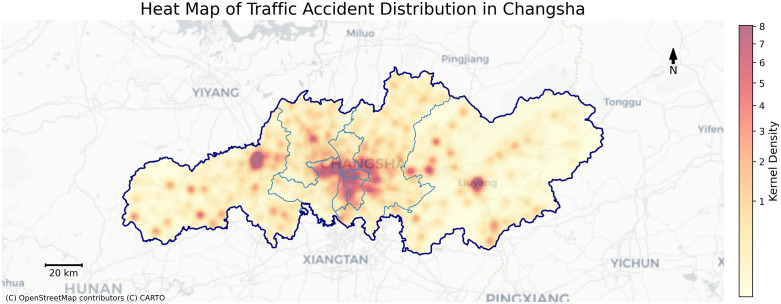
Heat map of traffic crashes in Changsha. Crash locations were obtained from the study dataset. The base map and data were obtained from OpenStreetMap and OpenStreetMap Foundation. This figure contains information from OpenStreetMap and OpenStreetMap Foundation, which is made available under the Open Database License. The heat map was created by the authors.

To further illustrate the spatial distribution of the crash samples, kernel density estimation was applied to visualize crash locations, and a traffic crash heat map of Changsha was generated (Fig 2). From the spatial pattern, crash samples showed a certain degree of clustering in urban built-up areas, along major arterial road corridors, and in zones with relatively concentrated functional activities. This indicates that traffic crash risk is not evenly distributed across urban space, but is spatially coupled with road network structure, built environment characteristics, and the intensity of traffic activity. This spatial pattern also provides a foundation for the subsequent analysis of traffic injury risk from the dual perspectives of 5D built environment factors and non-5D contextual factors.

### 3.2. Spatial matching and feature construction

Spatial matching was conducted to construct the built environment variables surrounding each crash location. First, crash records were geocoded as point features and matched with transit stop, POI, road network, and population density data. The distances to the nearest bus stop and metro station were calculated for each crash point to represent transit accessibility. For destination accessibility and land-use diversity, a 1,000 m buffer was generated around each crash point, within which the numbers of school, hospital, shopping mall, and tourist attraction POIs were counted. POI mix was calculated using the Shannon diversity index based on the composition of POI categories within the same buffer.

Road network density was calculated using a 1 km × 1 km grid. Each crash point was assigned to the grid cell in which it was located, and the total road length within each grid cell was divided by the grid area to obtain road network density. The 1 km × 1 km grid was selected primarily because the smallest available spatial resolution of the population density data used in this study was 1 km. Using the same grid resolution helped ensure consistency among population-related information, road network density, and crash locations. In addition, the 1 km grid provides a reasonable neighborhood-scale unit for capturing local road network structure while maintaining sufficient statistical stability. A much smaller spatial unit may introduce excessive local noise and may not be compatible with the available population density data, whereas a larger unit may obscure local built-environment heterogeneity.

### 3.3. Variable setting and feature grouping

In this study, traffic injury risk refers to injury occurrence conditional on crash occurrence. In other words, the proposed model predicts whether a recorded crash resulted in injury, rather than estimating the overall probability or frequency of injury crashes across the road network. The dependent variable was therefore defined as a binary outcome: a value of 1 was assigned if the crash caused personal injury, whereas a value of 0 was assigned if no injury occurred.

Although injury severity can also be analyzed using multiclass outcomes, such as fatal, serious, and slight injuries, the binary specification was adopted in this study for three main reasons. First, the primary aim of this study was to identify whether the surrounding built environment and crash-context conditions increase the likelihood that a crash crosses the fundamental threshold from non-injury to injury. This distinction is directly relevant to urban traffic safety screening and early risk intervention. Second, the explanatory variables in this study mainly characterize 5D built environment attributes and selected crash-context factors, which are more suitable for identifying spatial and contextual patterns of injury occurrence than for explaining detailed biomechanical injury severity mechanisms. Third, the mechanisms leading to fatal or severe injuries are often strongly associated with detailed crash dynamics, impact speed, collision configuration, vehicle damage, road-user behavior, restraint use, and post-crash medical outcomes, which were not fully available in the present dataset. Therefore, a binary injury occurrence model was considered more consistent with the research objective, data structure, and interpretability requirements of this study.

Compared with multiclass injury severity classification, this binary setting places greater emphasis on whether a crash results in human injury, and is therefore more consistent with the practical needs of traffic safety governance for the rapid identification and intervention of high-risk crash environments.

With respect to the explanatory variables, and in accordance with the research objectives and final modeling framework, the variables were divided into two categories: 5D variables and non-5D variables. Based on this classification, three feature groups were further constructed to compare the ability of different variable systems to identify traffic injury risk.

The 5D variables included median barrier, traffic signal control type, intersection/road segment type, road alignment, road type, distance to the nearest bus stop, distance to the nearest metro station, road network density, POI mix, and the numbers of schools, hospitals, shopping malls, and tourist attractions within a 1,000 m buffer. These variables were used to characterize the built environment surrounding the crash location from the perspectives of roadway design, transit accessibility, road network density, land-use mix, and destination accessibility.

By contrast, the non-5D variables included cross-sectional location, pavement condition, weather, lighting condition, day of week, and month. These variables mainly captured the immediate operating context, environmental status, and temporal attributes at the time of crash occurrence, thereby complementing the short-term dynamic influences that cannot be adequately represented by built environment variables alone.

Based on the above variables, three feature groups were constructed:

(1) 5D feature group, including only 5D variables;
(2) non-5D feature group, including only non-5D variables;
(3) 5D + non-5D feature group, in which both categories of variables were jointly entered into the model.

This grouping strategy not only helps examine whether 5D built environment factors can independently identify traffic injury risk, but also makes it possible to assess the extent to which the joint inclusion of 5D and non-5D factors improves model discriminative performance. In this study, the 5D variables were defined using an extended operational interpretation of the framework. In particular, the Design dimension was not limited to street network form alone, but was extended to include relatively stable roadway design, traffic-control infrastructure, and road functional characteristics that shape the physical and operational environment of crash locations. Median barriers were included because they represent roadway cross-sectional design and the separation of traffic streams. Traffic control type was included because signals, signs, and pavement markings constitute built traffic-control facilities that regulate road-user interactions and conflict patterns. Road type was included because it reflects the functional hierarchy, design standard, operating environment, and expected speed conditions of the roadway. Therefore, these variables were treated as built-environment-related roadway design and infrastructure variables within the extended 5D framework. In addition to the conventional dimensions of Density, Diversity, Destination accessibility, and Distance to transit, variables reflecting roadway spatial design and road environment organization were also incorporated into the Design dimension, so as to provide a more comprehensive characterization of the built environment surrounding crash locations.

Table 1 summarizes all explanatory variables included in the final models and their corresponding coding schemes. Continuous variables were entered in their original form, whereas categorical variables were encoded according to predefined rules. By explicitly linking the theoretical classification of variables with their actual model inputs, the table helps ensure consistency between the variable framework and the subsequent modeling procedures.

**Table 1:** Definition and coding of explanatory variables.

Category	Dimension/Attribute	Variable	Description of Coding	Coding
5D Variables	Design	Median Barrier	Absence of a median barrier	1
			Green belt	2
			Guardrail	3
5D Variables	Design	Traffic Control Type	No control	1
			Sign or pavement marking control	2
			Signal control	3
			Combined safety control	4
5D Variables	Design	Intersection/Segment Type	Mid-block segment	1
			Diverging intersection	2
			Bridge or tunnel segment	3
			Other special road segment	4
5D Variables	Design	Road Alignment	Straight segment	1
			Curved segment	2
			Grade segment	3
			Curved grade segment	4
5D Variables	Design	Road Type	Expressway	1
			Urban road	2
			Other	3
5D Variables	Distance to transit	Distance to the Nearest Bus Stop	Distance from the crash location to the nearest bus stop	Original value(m)
5D Variables	Distance to transit	Distance to the Nearest Metro Station	Distance from the crash location to the nearest metro station	Original value(m）
5D Variables	Density	Road Network Density	Road length within the 1 km × 1 km grid cell containing the crash location divided by grid cell area	Original value(km/km^2^)
5D Variables	Diversity	POI Mix	Shannon diversity index calculated based on surrounding POI categories	Original value
5D Variables	Destination accessibility	Number of Schools within 1,000 m	Number of school POIs within a 1,000 m buffer of the crash location	Original value
5D Variables	Destination accessibility	Number of Hospitals within 1,000 m	Number of hospital POIs within a 1,000 m buffer of the crash location	Original value
5D Variables	Destination accessibility	Number of Shopping Malls within 1,000 m	Number of shopping mall POIs within a 1,000 m buffer of the crash location	Original value
5D Variables	Destination accessibility	Number of Tourist Attractions within 1,000 m	Number of tourist attraction POIs within a 1,000 m buffer of the crash location	Original value
Non-5D Variables	Cross-sectional Location	Cross-sectional Location	Motor vehicle lane	1
			Mixed motor vehicle and non-motor vehicle lane	2
			Non-motor vehicle lane	3
			Sidewalk	4
			Other	5
Non-5D Variables	Pavement Condition	Pavement Condition	Dry	1
			Wet	2
			Waterlogged	3
			Snow/Ice	4
Non-5D Variables	Natural Environment	Weather	Weather conditions at the time of crash	Original coding
Non-5D Variables	Natural Environment	Lighting Condition	Lighting conditions at the time of crash	Original coding
Non-5D Variables	Temporal Attributes	Day of Week	Day of the week when the crash occurred	Original coding
Non-5D Variables	Temporal Attributes	Month	Month when the crash occurred	Original coding

### 3.4. Model construction and evaluation strategy

To systematically evaluate the performance of different variable systems and algorithms in identifying traffic injury risk, a dual analytical framework combining feature-group comparison and model comparison was adopted.

In the feature-group comparison, classification models were developed separately using the 5D feature group, the non-5D feature group, and the combined 5D + non-5D feature group, so as to examine both the independent and joint effects of different variable types on traffic injury risk discrimination. This design was intended to address two key questions: whether 5D variables alone could effectively identify traffic injury risk, and whether model performance could be further improved when 5D and non-5D variables were jointly incorporated.

In the model comparison, three algorithms—Random Forest (RF), eXtreme Gradient Boosting (XGBoost), and Categorical Boosting (CatBoost)—were implemented for each feature group. For RF and XGBoost, the training data were balanced using the Synthetic Minority Over-sampling Technique(SMOTE), followed by hyperparameter tuning. CatBoost, by contrast, was directly trained by leveraging its native capability to handle categorical features. Comparing multiple models in parallel helped reduce reliance on a single algorithm and improved the robustness of model selection.

### 3.5. Model training, imbalance handling, and evaluation

The dataset was divided into a training set and a testing set at a ratio of 8:2 using stratified random sampling, with a fixed random seed of 42 to ensure reproducibility. To address class imbalance, SMOTE was applied only to the training set after data splitting, while the testing set was kept unchanged to avoid information leakage.

For model comparison, RF, XGBoost, and CatBoost were implemented for each feature group. For RF and XGBoost, hyperparameter tuning was conducted on the training set using grid search combined with five-fold stratified cross-validation, with receiver operating characteristic-area under the curve (ROC-AUC) as the optimization criterion. CatBoost was trained without SMOTE by leveraging its native capability to handle categorical variables directly. Therefore, the final CatBoost model used for SHAP-based interpretation was not trained on synthetic samples generated by oversampling. This design helps ensure that the interpretation of key influencing factors is not dependent on SMOTE-generated observations.

Model performance was evaluated using accuracy, precision, recall, F1-score, and ROC-AUC. In the robustness check related to imbalance handling, specificity was additionally reported to assess the models’ ability to identify non-injury crashes. The candidate parameter ranges and final hyperparameter settings for the models under the combined 5D + non-5D feature group are reported in S3 Table.

### 3.6. Random Forest and XGBoost models

Random Forest (RF) is an ensemble learning method based on bagging and decision trees. It constructs multiple decision trees using bootstrap samples and random subsets of predictors, and the final classification result is obtained by aggregating the predictions of all trees. RF can capture nonlinear relationships and variable interactions, and is relatively robust to noise and overfitting. In this study, RF was used as a representative bagging-based ensemble model for comparison with boosting-based models.

eXtreme Gradient Boosting (XGBoost) is a gradient boosting decision tree model that builds trees sequentially, with each new tree fitted to reduce the residual errors of the previous trees. Compared with conventional boosting methods, XGBoost incorporates regularization, shrinkage, column subsampling, and efficient tree optimization, which can improve predictive performance and reduce overfitting. In this study, XGBoost was used as a representative boosting-based model and compared with RF and CatBoost to evaluate the performance of different ensemble learning strategies in identifying injury occurrence among recorded crashes.

### 3.7. CatBoost model

Categorical Boosting (CatBoost) is an ensemble learning method developed within the gradient boosting decision tree framework. Its primary advantage lies in its ability to handle categorical variables directly while effectively capturing nonlinear relationships and high-order interactions among variables. For the traffic crash dataset used in this study, categorical variables account for a substantial proportion of the predictors. Variables such as median barrier, traffic control type, intersection/segment type, road alignment, road type, cross-sectional location, pavement condition, weather, and lighting condition are all discrete features. CatBoost therefore shows clear methodological suitability for the present analysis.

Compared with conventional gradient boosting decision tree (GBDT) methods, CatBoost adopts ordered target statistics and an ordered boosting mechanism for categorical feature processing, thereby effectively reducing target leakage and prediction shift and improving the model’s generalization ability. For binary classification tasks, CatBoost iteratively constructs weak learners and combines them through weighted aggregation, allowing the overall model to gradually approach the optimal classification boundary. The updating process can be expressed as follows:


$$\begin{array}{c} F_{t}(x)=F_{t-\text{1}}(x)+\eta h_{t}(x) \end{array}$$ (1)

where$\;F_{t}(x)$ is the model output after the t-th iteration, $h_{t}(x)$is the decision tree generated at iteration t, and η denotes the learning rate. The model progressively improves classification performance by fitting the residuals or negative gradients from the previous iteration.

CatBoost was ultimately selected as the final model not only because it delivered the best overall discriminative performance under the combined feature group, but also because it is able to process a large number of categorical variables directly, thereby avoiding the dimensional expansion and potential information loss caused by one-hot encoding. Moreover, CatBoost is highly compatible with interpretability methods such as SHAP, which provides a robust technical basis for identifying key influencing factors and explaining their underlying mechanisms in the subsequent analysis.

Although tree-based models such as XGBoost and CatBoost can provide model-intrinsic feature importance measures, these measures usually summarize the average contribution of variables to tree splitting, gain, or prediction loss reduction. They are useful for identifying important predictors at the global level, but they provide limited information on the direction, magnitude, and heterogeneity of variable effects. In particular, conventional feature importance measures do not directly show whether higher or lower values of a variable increase the probability of injury occurrence, nor do they reveal nonlinear response patterns across different value ranges.

Therefore, SHAP was further employed in this study to provide a more detailed and interpretable explanation of the final CatBoost model. Based on Shapley values, SHAP decomposes each model prediction into additive contributions from individual variables, thereby allowing both global feature ranking and local interpretation at the observation level. Compared with conventional tree-based feature importance, SHAP provides additional insights into the direction of influence, the distribution of variable effects across samples, nonlinear dependence patterns, and the relative contribution of 5D and non-5D variables. These advantages make SHAP particularly suitable for interpreting how built environment and crash-context factors jointly influence injury occurrence among recorded crashes.

## 4. Results

### 4.1 Model comparison results

Table 2 presents the classification results for different feature groups and models. Overall, substantial differences were observed in the predictive performance of the different variable systems, and the three models did not perform consistently across the feature groups.

**Table 2:** Comparative Classification Performance across Different Feature Groups and Models.

Feature group	Model	accuracy	precision	recall	f1	roc_auc
Combined 5D + non-5D	RF	0.939456	0.953769	0.980758	0.967076	0.926009
Combined 5D + non-5D	CatBoost	0.938430	0.946341	0.988115	0.966777	0.941496
Non-5D	CatBoost	0.935351	0.942318	0.989247	0.965213	0.924083
Combined 5D + non-5D	XGBoost	0.935351	0.953566	0.976231	0.964765	0.929375
Non-5D	XGBoost	0.928168	0.953203	0.968308	0.960696	0.922845
Non-5D	RF	0.924577	0.952009	0.965478	0.958696	0.916222
5D	CatBoost	0.912263	0.918678	0.990945	0.953444	0.773834
5D	RF	0.869164	0.940047	0.913978	0.926829	0.769571
5D	XGBoost	0.863520	0.931074	0.917374	0.924173	0.727510

From Table 2, clear differences were observed in the discriminative performance of the three feature groups. When only the 5D feature group was used, the overall performance of the three models was relatively limited. Among them, CatBoost achieved an accuracy of 0.912263, a recall of 0.990945, an F1-score of 0.953444, and a ROC-AUC of 0.773834, while the ROC-AUC values of RF and XGBoost were 0.769571 and 0.727510, respectively. These results indicate that although the 5D variables were able to identify a portion of injury-related crash samples, their overall ability to discriminate between positive and negative samples remained limited.

When only the non-5D feature group was used, model performance improved markedly. For non-5D CatBoost, the accuracy, recall, F1-score, and ROC-AUC reached 0.935351, 0.989247, 0.965213, and 0.924083, respectively. The ROC-AUC values of non-5D XGBoost and non-5D RF were 0.922845 and 0.916222, respectively. Compared with the 5D feature group, the non-5D feature group consistently achieved better performance across all evaluation metrics.

When the combined 5D + non-5D feature group was adopted, model performance improved further. Specifically, 5D + non-5D RF achieved the highest accuracy (0.939456), although its ROC-AUC was 0.926009. 5D + non-5D XGBoost yielded a ROC-AUC of 0.929375. By comparison, 5D + non-5D CatBoost achieved an accuracy of 0.938430, a precision of 0.946341, a recall of 0.988115, an F1-score of 0.966777, and the highest ROC-AUC of 0.941496 among all models. Although RF achieved a slightly higher accuracy under the combined feature group, CatBoost produced the strongest threshold-independent discriminative performance, as reflected by ROC-AUC, while maintaining competitive accuracy, precision, recall, and F1-score.

ROC-AUC was used as the primary model-selection criterion because it evaluates the model’s ability to distinguish injury from non-injury crashes across all possible classification thresholds and is less dependent on a specific decision threshold than accuracy or recall. This is particularly important given the imbalanced outcome distribution of the crash data. Therefore, CatBoost was selected as the benchmark model for subsequent SHAP-based interpretation based on its overall balance of ROC-AUC, classification performance, ability to directly handle categorical variables, and compatibility with SHAP analysis.

It should be noted that the improvement in accuracy from the non-5D CatBoost model to the combined 5D + non-5D CatBoost model was relatively small, increasing from 0.935351 to 0.938430. This indicates that the addition of 5D variables contributed only a limited improvement in accuracy. However, the ROC-AUC increased from 0.924083 to 0.941496, suggesting that 5D variables still provided additional threshold-independent discriminative information when combined with non-5D contextual factors. Therefore, the contribution of 5D variables should be interpreted cautiously: non-5D variables showed strong standalone predictive performance, whereas 5D variables provided complementary spatial-environmental information and contributed substantially to the model-based SHAP explanation within the combined CatBoost model.

To further examine whether the high recall values were driven by SMOTE, an additional robustness check was conducted for the combined 5D + non-5D feature group under different imbalance-handling strategies (S1 Table). The results showed that high recall values were also observed in models trained without SMOTE. For example, RF without SMOTE achieved a recall of 0.994907, which was comparable to that of RF with SMOTE. This suggests that the high recall values were not solely caused by synthetic samples generated by SMOTE, but were also related to the imbalanced distribution of the original testing set. In addition, the class-weight-adjusted XGBoost model achieved a much higher specificity of 0.631868, although its recall decreased to 0.838144. This result indicates a trade-off between sensitivity to injury crashes and discrimination of non-injury crashes. Therefore, the high recall values should be interpreted as strong sensitivity to injury crashes rather than as uniformly strong classification performance across both classes, and model performance should be evaluated using multiple metrics rather than accuracy and recall alone. It should be noted that S1 Table was designed as a sensitivity analysis under alternative imbalance-handling strategies and was not intended to reproduce the main model comparison results in Table 2.

To examine whether spatial dependence remained in the final model, Moran’s I was calculated for the residuals of the CatBoost model using an 8-nearest-neighbor spatial weight matrix and 999 permutations. As shown in S2 Table, Moran’s I was 0.073620, with an expected value of −0.000513 and a pseudo p-value of 0.001. This result indicates that the residuals still exhibited statistically significant but relatively weak positive spatial autocorrelation. Therefore, the SHAP-based importance of location-based 5D variables should be interpreted as model-based relative contributions rather than spatially unbiased causal effects.

### 4.2. SHAP analysis of overall contributions

After CatBoost was selected as the optimal model, SHAP (Shapley Additive Explanations) was further applied to interpret the model outputs and to identify differences in the contributions of variable categories and individual features to traffic injury risk. For clarity, only the top 10 ranked variables were displayed in the SHAP plots.

**Fig 3:**
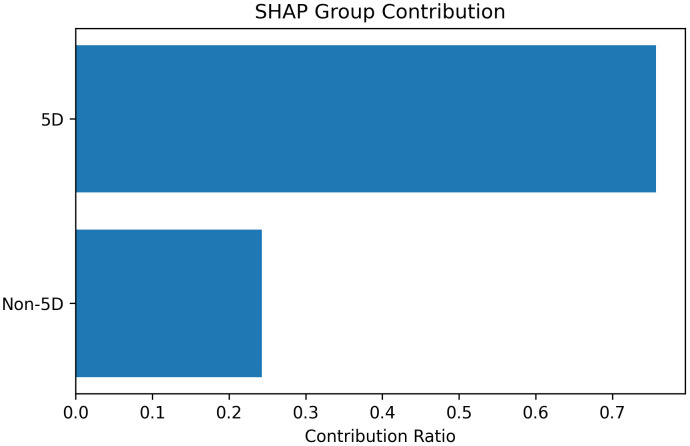
SHAP group-level contributions.

As shown in Fig 3, the 5D variables contributed approximately 75% of the total SHAP value, whereas the non-5D variables accounted for roughly 25%. This result suggests that the cumulative explanatory contribution of the 5D variables in the combined model was substantially greater than that of the non-5D variables. In other words, although the model comparison indicated that the non-5D feature group exhibited relatively strong predictive performance when used alone, built-environment-related variables contributed more information and exerted a stronger influence on model outputs within the joint interpretive framework.

To further identify the key determinants of traffic injury risk, the SHAP values of the optimal CatBoost model were summarized at the global level to generate the feature importance ranking.

**Fig 4:**
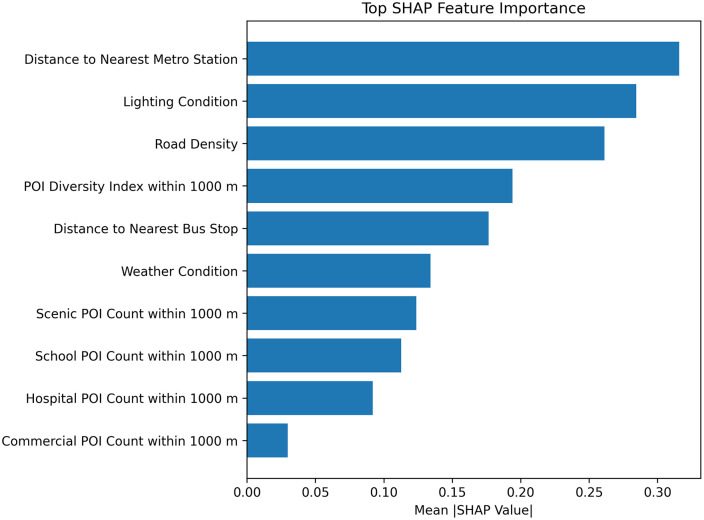
SHAP feature importance.

As shown in Fig 4, the top 10 variables were, in descending order of importance, distance to the nearest metro station, lighting condition, road network density, POI mix within 1,000 m, distance to the nearest bus stop, weather condition, number of tourist attractions within 1,000 m, number of schools within 1,000 m, number of hospitals within 1,000 m, and number of shopping malls within 1,000 m. Among these variables, distance to the nearest metro station had the largest mean absolute SHAP value, indicating that it exerted the strongest marginal effect on traffic injury risk and served as the most influential explanatory variable in the current model.

From the perspective of variable type, eight of the top 10 variables belonged to the 5D category, whereas only lighting condition and weather condition were classified as non-5D variables. This finding is consistent with the previous group-level contribution analysis and further suggests that 5D variables not only dominated in terms of total contribution, but also occupied a leading position in the ranking of key features.

In terms of variable attributes, the most important factors were mainly concentrated in four aspects. The first was transit accessibility, as reflected in the high importance of both distance to the nearest metro station and distance to the nearest bus stop. The second was spatial structure, represented by road network density and POI mix. The third was environmental and operational conditions, mainly captured by lighting condition and weather condition. The fourth was destination accessibility, reflected in the numbers of schools, hospitals, tourist attractions, and shopping malls.

Based on the global feature importance ranking, the SHAP beeswarm plot was further used to examine the direction of variable effects and their heterogeneous patterns.

**Fig 5:**
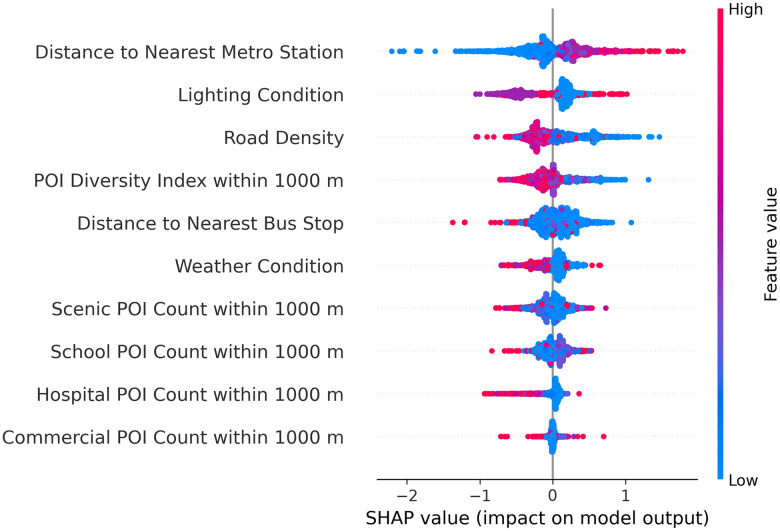
SHAP beeswarm plot.

As shown in Fig 5, the distributions of SHAP values were asymmetric across variables, and substantial differences were observed in the degree of dispersion among features, indicating pronounced nonlinearity and individual heterogeneity in their effects on traffic injury risk.

For distance to the nearest metro station, observations with higher values were mainly distributed in the positive SHAP region, whereas lower-value observations were more concentrated around zero or in the negative SHAP region. This pattern suggests that as crash locations become farther from metro stations, traffic injury risk tends to increase; conversely, better metro accessibility is more likely to reduce injury risk. A similar pattern was observed for distance to the nearest bus stop, where areas with poorer bus stop accessibility were more likely to exhibit positive SHAP values and, in turn, a higher injury risk.

Road network density and POI mix showed directional patterns different from those of the above accessibility variables. Higher road network density and greater POI mix were more frequently associated with negative SHAP values, indicating a suppressing effect on traffic injury risk. By contrast, lower road network density and lower POI mix were more likely to shift the model output toward injury occurrence.

Although lighting condition and weather condition belonged to the non-5D category, they still exhibited relatively high importance and a wide spread of points in the beeswarm plot, suggesting that they had substantial effects on traffic injury risk.

Although the numbers of schools, hospitals, tourist attractions, and shopping malls were less important overall than transit accessibility and road network characteristics, all four variables were ranked among the top 10 features. This result indicates that destination concentration also provides a non-negligible explanation for traffic injury risk.

### 4.3. Nonlinear effects of key continuous variables

**Fig 6:**
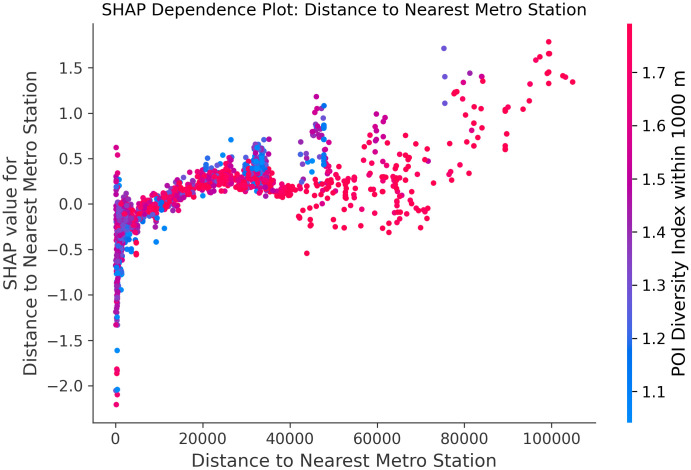
SHAP dependence plot for distance to the nearest metro station.

As shown in Fig 6, a clear nonlinear relationship was observed between distance to the nearest metro station and the corresponding SHAP value. Overall, the SHAP value exhibited an upward trend as the distance increased, indicating that crash locations farther from metro stations exerted a stronger positive effect on traffic injury risk. In particular, within the shorter distance range, SHAP values were mostly concentrated in the negative region. As the distance increased further, however, the SHAP values gradually shifted to the positive region and continued to rise, suggesting that reduced metro accessibility substantially increased traffic injury risk.

**Fig 7:**
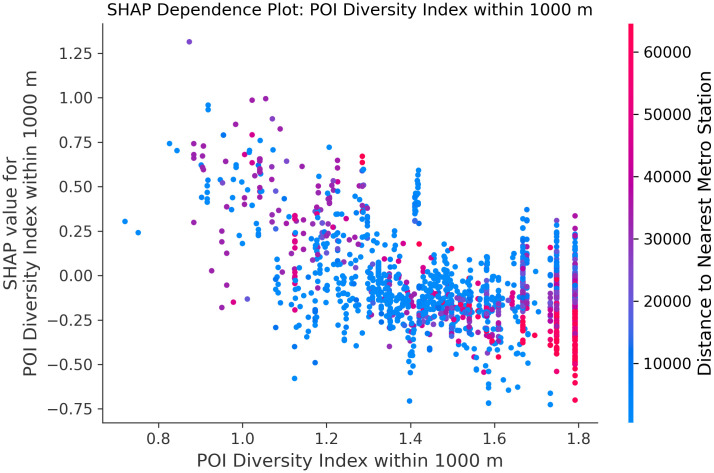
SHAP dependence plot for POI mix Index within 1000 m.

As shown in Fig 7, a relatively clear negative relationship was observed between POI mix and the corresponding SHAP value. As POI mix increased from lower to higher levels, the SHAP value gradually shifted from positive to negative, indicating that a higher degree of functional mix generally helped reduce traffic injury risk.

**Fig 8:**
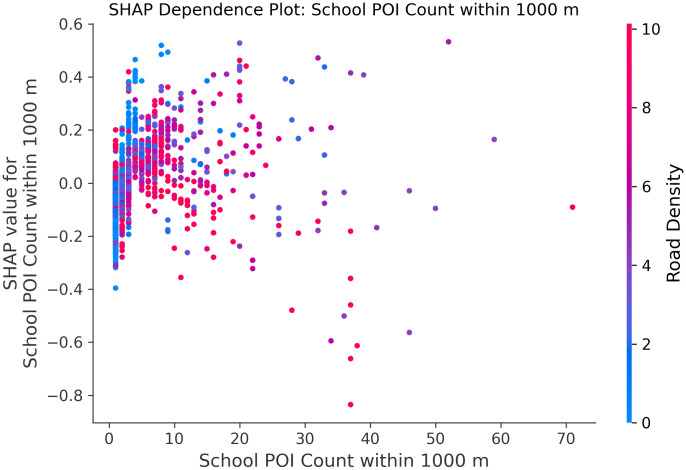
SHAP dependence plot for School POI Count within 1000 m.

As shown in Fig 8, the relationship between the number of schools within 1,000 m and the corresponding SHAP value was not monotonic or linear, but instead exhibited noticeable fluctuations. Within the low-to-moderate range of school counts, SHAP values could be either positive or negative. Overall, however, the magnitude of the variable’s effect on model output became more pronounced as the number of schools increased, suggesting that the influence of school concentration on traffic injury risk was highly context-dependent.

## 5. Discussion

### 5.1. Joint effects of 5D and non-5D factors

The results of this study showed that the combined 5D + non-5D feature group consistently outperformed the models built with either the 5D or non-5D feature group alone, indicating that traffic injury risk is shaped not by a single category of determinants, but by the interaction between the spatial built environment and the immediate crash context. An important finding is that the non-5D feature group demonstrated relatively strong predictive performance when modeled independently, whereas the SHAP group-level contribution analysis indicated that 5D variables accounted for a substantially larger cumulative contribution in the combined model. These findings should not be interpreted as contradictory. Rather, they reflect two different dimensions of model understanding: These findings should be interpreted by distinguishing predictive performance from model-based explanation. The non-5D feature group achieved strong standalone performance, suggesting that immediate crash-context factors have substantial direct discriminative value for distinguishing injury from non-injury crashes. The addition of 5D variables produced only a marginal improvement in accuracy, indicating that their incremental contribution to overall predictive accuracy was limited. Nevertheless, the increase in ROC-AUC and the SHAP results suggest that 5D variables provided complementary spatial-environmental information and contributed substantially to the internal explanation of the combined CatBoost model. Therefore, the 5D variables should not be interpreted as the primary source of predictive performance, but rather as an important component for explaining spatial and built-environment-related heterogeneity in injury risk.

This pattern suggests that traffic injury risk is characterized by both immediate situational sensitivity and spatial embeddedness. On the one hand, variables such as weather, lighting condition, and pavement status directly influence visibility, vehicle handling, and conflict severity at the moment of crash occurrence, thereby exerting an immediate effect on injury outcomes. On the other hand, 5D variables—including transit accessibility, roadway design, road network density, and destination distribution—more fundamentally shape the spatial organization, activity intensity, and exposure structure of the crash environment. In this sense, non-5D factors may function more as proximate triggers of injury occurrence, whereas 5D factors provide the underlying contextual conditions within which injury risk is generated and differentiated.

### 5.2. Dominant role of transit accessibility and spatial structure

Among all variables, distance to the nearest metro station, distance to the nearest bus stop, road network density, and POI mix exhibited particularly high importance, indicating that transit accessibility and spatial structure play central roles in shaping traffic injury risk. Better public transport accessibility often reflects more mature roadway infrastructure, higher-quality street environments, and more organized traffic operations. Similarly, higher road network density and stronger functional mix are typically associated with finer-grained street networks, shorter travel distances, and a more balanced distribution of urban activities. Taken together, these characteristics may influence not only exposure patterns and conflict opportunities, but also the likelihood that a crash escalates into an injury event.

The association between proximity to metro stations and lower injury risk may be explained through several complementary mechanisms. First, metro station areas usually concentrate a large number of pedestrians and transit users. This may generate a “safety in numbers” effect, whereby higher pedestrian presence increases drivers’ awareness and encourages more cautious driving behavior. Second, areas with better metro accessibility are often characterized by transit-oriented and compact urban development, including denser street networks, more frequent intersections, shorter blocks, lower automobile dependence, and more complete pedestrian facilities. These built environment characteristics may contribute to lower vehicle operating speeds and reduce the probability that a crash results in injury.

However, this interpretation should be made cautiously. The present study did not include direct measures of pedestrian volume, traffic flow, or vehicle operating speed. Therefore, it is not possible to determine whether the observed association is mainly attributable to a “safety in numbers” effect, lower vehicle speeds in transit-oriented environments, or the combined influence of both mechanisms. Accordingly, proximity to metro stations should be interpreted as a location-based indicator associated with safer and more organized urban traffic environments, rather than as a direct causal factor reducing injury risk.

At the same time, the numbers of schools, hospitals, tourist attractions, and shopping malls were all ranked among the top 10 variables, suggesting that destination accessibility captures more than the intensity of local activities alone. It may also affect injury outcomes by reshaping the interaction patterns among pedestrians, non-motorized users, and motor vehicles, thereby altering the frequency and severity of potential traffic conflicts.

### 5.3. Comparison with previous studies

The findings of this study should be interpreted in relation to previous literature on the built environment and traffic safety. Previous studies have suggested that built environment characteristics may influence traffic safety through multiple pathways, including crash exposure, crash frequency, and crash severity or injury outcomes [7–12,25]. For example, Saha et al. [8] proposed that the built environment affects traffic safety indirectly through exposure, conflict frequency, and operating speed, while Merlin et al. [9] emphasized the importance of distinguishing crash exposure from crash risk. Asadi et al. [12] further showed that built environment factors are associated with crash probability, frequency, and severity, and Sung et al. [25] found that compact and mixed-use environments may have different associations with pedestrian crash occurrence and fatal crash risk.

However, the present study does not directly analyze crash frequency. Instead, it focuses on whether a recorded crash resulted in injury, that is, injury occurrence conditional on crash occurrence. Within this conditional framework, variables related to transit accessibility, road network structure, and functional mix showed important model-based explanatory contributions. Therefore, the present findings should be understood as evidence that built environment factors are associated with injury outcomes among recorded crashes, rather than as direct evidence that these factors affect the overall frequency of injury crashes.

It should be noted that the SHAP-based feature importance reflects the relative contribution of variables within the fitted CatBoost model. The Moran’s I test of model residuals indicated statistically significant but relatively weak positive spatial autocorrelation, suggesting that some spatial dependence was not fully captured by the current feature set. Therefore, the importance of location-based 5D variables, such as distance to transit, road network density, and POI mix, may partly reflect spatially clustered built environment characteristics. These results should therefore be interpreted as model-based associations rather than causal spatial effects.

### 5.4. Practical implications

From a practical perspective, the findings of this study suggest that traffic safety governance should not be limited to reactive responses after crashes occur, nor should it rely solely on isolated roadway engineering measures. Greater emphasis should instead be placed on the coordinated optimization of the built environment surrounding crash locations and the immediate contextual conditions under which crashes occur. On the one hand, regional spatial structure should be improved through better transit stop placement, road network organization, functional mix, and destination configuration. On the other hand, safety management and risk warning systems should be strengthened under immediate high-risk conditions related to lighting, weather, and pavement status. Only by integrating spatial environment optimization with contextual risk control can the likelihood that a crash develops into an injury outcome be reduced more effectively.

## 6. Conclusions

Based on traffic crash data from Changsha, China, covering the period from 2017 to 2019, this study developed an analytical framework that integrates 5D built environment variables and non-5D contextual variables to examine traffic injury risk, defined here as injury occurrence conditional on a recorded crash. Therefore, the proposed model should be interpreted as predicting injury occurrence conditional on crash occurrence, rather than the overall probability or frequency of injury crashes across the road network. Within this framework, RF, XGBoost, and CatBoost were employed to compare the performance of traffic injury risk identification, and the SHAP method was further applied to reveal the key influencing factors and their effect patterns.

The results yielded four main findings. First, the combined 5D + non-5D feature group consistently outperformed the models using either the 5D or non-5D feature group alone, indicating that traffic injury risk is not determined by a single category of factors, but rather by the joint effects of built environment characteristics and immediate contextual conditions. Second, among the three models, CatBoost achieved the best overall performance under the combined feature group. Its accuracy, recall, and F1-score all remained at relatively high levels, and it produced the highest ROC-AUC value, suggesting superior overall discriminative ability and robustness in traffic injury risk identification. Third, the SHAP analysis showed that 5D variables accounted for a larger share of the model-based contribution within the combined CatBoost model. However, because the improvement in accuracy from the non-5D model to the combined model was relatively small, this result should be interpreted as indicating that 5D variables provide complementary explanatory information rather than serving as the dominant source of predictive performance. Fourth, distance to the nearest metro station, lighting condition, road network density, POI mix, and distance to the nearest bus stop were identified as the most influential factors affecting traffic injury risk, and the key continuous variables exhibited pronounced nonlinear and heterogeneous effects.

Overall, from the coupled perspective of 5D built environment characteristics and non-5D contextual factors, this study reveals the multidimensional pathways through which traffic injury risk is shaped and demonstrates the effectiveness of the CatBoost–SHAP framework for both identifying and interpreting traffic injury risk. The findings provide empirical support for urban traffic safety risk identification, roadway environment optimization, and refined people-oriented traffic safety governance.

## 7. Limitations and future research

Despite the contributions of this study, several limitations should be acknowledged. First, the analysis was based solely on traffic crash data from Changsha for the period 2017–2019. As a result, the findings may have been influenced by the city’s specific spatial structure, traffic management practices, and temporal context, and their external generalizability should be further examined using data from additional cities and longer time periods.

Second, crash outcome was modeled as a binary variable indicating whether injury occurred. Although this specification is consistent with the main objective of identifying injury-prone crash environments, it does not distinguish among different injury severity levels, such as slight injury, serious injury, and fatality. We acknowledge that the mechanisms leading to fatal or severe injuries may differ substantially from those leading to minor injuries. In particular, higher-level injury outcomes are often influenced by detailed crash dynamics, impact speed, collision configuration, vehicle damage, road-user characteristics, restraint use, and post-crash emergency response, which were not fully captured in the present dataset. Therefore, the findings of this study should be interpreted as evidence on injury occurrence rather than on the full severity spectrum. Future research should extend the proposed framework to multiclass, ordered, or hierarchical severity models when more detailed crash-mechanism and injury-severity data become available.

Third, although this study incorporated several location-based 5D built environment variables, it did not explicitly model spatial autocorrelation among crash observations. The Moran’s I test of CatBoost model residuals indicated statistically significant but relatively weak positive spatial autocorrelation, suggesting that some residual spatial dependence remained. Therefore, the estimated importance of location-based variables, such as distance to transit, road network density, and POI mix, may be affected by spatially clustered built environment characteristics or other unobserved spatial factors. In this study, SHAP values should be interpreted as model-based relative contributions under the current feature set rather than as spatially unbiased causal effects. Future research should further incorporate spatial econometric models, geographically weighted models, spatial random effects, spatially blocked cross-validation, or geospatial machine learning approaches to better account for spatial dependence and spatial heterogeneity.

Although this study conducted imbalance-handling sensitivity analysis and residual spatial autocorrelation diagnostics, repeated cross-validation and spatially blocked cross-validation were not fully implemented. Future research should further apply repeated or spatially blocked cross-validation to evaluate the robustness and spatial transferability of the proposed model across different spatial partitions.

In addition, although proximity to metro stations was associated with lower injury risk, the dataset did not contain direct measures of pedestrian volume, vehicle operating speed, or traffic flow. Therefore, this study could not empirically distinguish whether this association was driven by a “safety in numbers” effect, lower operating speeds in transit-oriented environments, or other unobserved characteristics of metro station areas.

Although weather condition was included as a non-5D contextual variable, this study did not incorporate long-term climate change indicators, extreme weather measures, or dynamic traffic flow density due to data availability limitations. Future research should integrate meteorological data, climate-related indicators, and real-time or aggregated traffic flow variables to further examine how natural environmental conditions and traffic operational states interact with built environment factors in shaping injury occurrence among recorded crashes.

## Supporting information

S1 Table Sensitivity analysis of RF and XGBoost performance under alternative imbalance-handling strategies for the combined 5D + non-5D feature group. Note: S1 Table was designed as a sensitivity analysis under alternative imbalance-handling strategies and was not intended to reproduce the main model comparison results in Table 2.
(DOCX)

S2 Table Moran’s I test for spatial autocorrelation of CatBoost model residuals. Note: Moran’s I was calculated for the residuals of the final CatBoost model using an 8-nearest-neighbor spatial weight matrix and 999 permutations. K-neighbors indicates the number of nearest neighbors used to construct the spatial weight matrix.
(DOCX)

S3 Table Candidate ranges and final hyperparameter settings for RF, XGBoost, and CatBoost under the combined 5D + non-5D feature group. (DOCX)
