# Supplementary material for: Effects of 5D built environment and non-built-environment factors on injury crash risk: An interpretable machine learning analysis
Source: PLoS One. 2026 Jul 7;21(7):e0353205. doi: 10.1371/journal.pone.0353205 (PMC13340810; doi:10.1371/journal.pone.0353205)
Supplement: S1 Table — Note: S1 Table was designed as a sensitivity analysis under alternative imbalance-handling strategies and was not intended to reproduce the main model comparison results in Table 2. (DOCX) [file pone.0353205.s001.docx]

| **Model** | **Imbalance handling** | **Accuracy** | **Precision** | **Recall** | **F1** | **ROC-AUC** | **Specificity** |
| --- | --- | --- | --- | --- | --- | --- | --- |
| RF | SMOTE sensitivity setting | 0.905593 | 0.919005 | 0.982456 | 0.949672 | 0.804614 | 0.159341 |
| RF | No SMOTE | 0.913802 | 0.917058 | 0.994907 | 0.954397 | 0.829384 | 0.126374 |
| XGBoost | SMOTE sensitivity setting | 0.908671 | 0.922831 | 0.981324 | 0.951179 | 0.806532 | 0.203297 |
| XGBoost | Class-weight adjustment | 0.818881 | 0.956718 | 0.838144 | 0.893514 | 0.795052 | 0.631868 |
