# Supplementary material for: Effects of 5D built environment and non-built-environment factors on injury crash risk: An interpretable machine learning analysis
Source: PLoS One. 2026 Jul 7;21(7):e0353205. doi: 10.1371/journal.pone.0353205 (PMC13340810; doi:10.1371/journal.pone.0353205)
Supplement: S2 Table — Note: Moran’s I was calculated for the residuals of the final CatBoost model using an 8-nearest-neighbor spatial weight matrix and 999 permutations. K-neighbors indicates the number of nearest neighbors used to construct the spatial weight matrix. (DOCX) [file pone.0353205.s002.docx]

| **Moran’s I** | **Expected I** | **Pseudo p-value** | **N** | **K-neighbors** | **Permutations** |
| --- | --- | --- | --- | --- | --- |
| 0.073620 | -0.000513 | 0.001 | 1949 | 8 | 999 |
