# Supplementary material for: Effects of 5D built environment and non-built-environment factors on injury crash risk: An interpretable machine learning analysis
Source: PLoS One. 2026 Jul 7;21(7):e0353205. doi: 10.1371/journal.pone.0353205 (PMC13340810; doi:10.1371/journal.pone.0353205)
Supplement: S3 Table — (DOCX) [file pone.0353205.s003.docx]

| **Feature group** | **Model** | **Hyperparameter** | **Candidate range** | **Final setting** |
| --- | --- | --- | --- | --- |
| Combined 5D + non-5D | RF | n_estimators | 200, 300 | 300 |
| Combined 5D + non-5D | RF | max_depth | None, 10, 15 | None |
| Combined 5D + non-5D | RF | min_samples_split | 2, 5 | 2 |
| Combined 5D + non-5D | RF | min_samples_leaf | 1, 2 | 1 |
| Combined 5D + non-5D | XGBoost | n_estimators | 200, 300 | 300 |
| Combined 5D + non-5D | XGBoost | max_depth | 4, 6 | 6 |
| Combined 5D + non-5D | XGBoost | learning_rate | 0.03, 0.05, 0.10 | 0.10 |
| Combined 5D + non-5D | XGBoost | subsample | 0.8, 1.0 | 0.8 |
| Combined 5D + non-5D | XGBoost | colsample_bytree | 0.8, 1.0 | 1.0 |
| Combined 5D + non-5D | CatBoost | iterations | Fixed | 300 |
| Combined 5D + non-5D | CatBoost | depth | Fixed | 6 |
| Combined 5D + non-5D | CatBoost | learning_rate | Fixed | 0.05 |
| Combined 5D + non-5D | CatBoost | loss_function | Fixed | Logloss |
| Combined 5D + non-5D | CatBoost | eval_metric | Fixed | AUC |
| Combined 5D + non-5D | CatBoost | random_seed | Fixed | 42 |
